# Editorial: Autoimmune diseases: from molecular mechanisms to therapy development

**DOI:** 10.3389/fimmu.2025.1768955

**Published:** 2026-01-06

**Authors:** Patrizia Leone, Nicola Susca, Vito Racanelli

**Affiliations:** 1 Department of Interdisciplinary Medicine, Aldo Moro University of Bari, Bari, Italy; 2 Internal Medicine Division, Santa Chiara Hospital, Provincial Health Care Agency, (APSS), Trento, Italy; 3 Centre for Medical Sciences, University of Trento and Internal Medicine Division, Santa Chiara Hospital, Provincial Health Care Agency (APSS), Trento, Italy

**Keywords:** antibody, autoimmune disease, immune tolerance, microenvironment, therapeutic strategies

## 1 Introduction

Autoimmune diseases comprise a heterogeneous group of disorders characterized by the immune system’s failure to distinguish between self and non-self, leading to aberrant immune responses that target the body’s own cells and tissues. This dysregulation manifests as chronic inflammation, tissue damage, and systemic dysfunction, with clinical presentations ranging from severe organ failure to subtle biochemical abnormalities. The pathogenesis of autoimmune diseases is shaped by complex interactions among genetic predisposition, environmental triggers, and epigenetic modifications, which together undermine immune self-tolerance.

Given their wide-ranging effects on multiple organ systems and the overlap in clinical manifestations, diagnosing autoimmune disorders is inherently challenging. Proper diagnosis requires the identification of disease-specific biomarkers and the performance of tests to rule out other conditions.

This Research Topic delves into the latest advances in understanding the molecular and cellular mechanisms underlying autoimmunity and current therapeutic approaches. These range from broadly used anti-inflammatory drugs to recent molecular-targeted therapies, such as Janus kinase (JAK) inhibitors, monoclonal antibodies, and emerging cellular therapies, including chimeric antigen receptor (CAR) T cell therapy and regulatory T cell (Treg) adoptive transfer or induction (Horwitz et al.).

## 2 The underlying mechanisms of autoimmune diseases

At the core of autoimmune diseases lies the breakdown of immune self-tolerance, a process that drives the activation of autoreactive immune cells, especially T cells and B cells (1). In these diseases, an imbalance arises between effector T cells and Tregs, both locally at the site of tissue damage and systemically. The depletion or functional alteration of Tregs allows autoreactive cytotoxic CD8^+^ T cells to operate freely. They can migrate into affected tissues and directly interact with and destroy target cells. Additionally, CD4^+^ T cells exacerbate tissue damage by releasing large amounts of pro-inflammatory cytokines or activating B cells. These inflammatory signals draw many myeloid immune cells to the site of tissue injury. This initiates a localized immune response. Activated B cells mature into plasma cells, producing significant quantities of antibodies against self-antigens. These autoantibodies can activate the complement system, mediate cell destruction through antibody-dependent cell-mediated cytotoxicity (ADCC), or form immune complexes with various nucleic acids and related proteins. Immune complexes can deposit and trigger a strong inflammatory response, damaging organs and tissues. For example, in rheumatoid arthritis (RA), they contribute to osteocyte death and osteoarticular damage (2). In systemic lupus erythematosus (SLE), these complexes target the kidneys, leading to immune-mediated glomerulonephritis (2).

Furthermore, the inflammatory response in autoimmune diseases is self-sustaining, driven by the release of pro-inflammatory cytokines and other signaling molecules (2). Notably, the Janus kinase-signal transducer and activator of transcription (JAK/STAT) pathway plays a pivotal role in sustaining this inflammatory cascade by regulating the expression of key pro-inflammatory genes. Dysregulation of the JAK/STAT pathway may promote the immune-mediated destruction of pancreatic beta-cells in type 1 diabetes (T1D) (Su et al.) and the intestinal epithelial cell death, along with intestinal barrier problems in inflammatory bowel disease (IBDs) in both humans and mice (Akanyibah et al.).

The interplay between the innate and specific arms of immunity is complex and multifaceted, as illustrated by the case presented by Smertinaite et al., in which an inflammatory stimulus, such as ectopic pregnancy, drives the relapse of myelin oligodendrocyte glycoprotein antibody-associated disease (MOGAD). The authors demonstrate that the relapse was associated with increased IL-1 and that a potent B-cell-depleting therapy can hamper this process.

Chronic overactivation of plasmacytoid dendritic cells through Toll-like receptors 7 and 9 and the cGAS–STING signaling pathway leads to sustained type I interferon production during inflammatory responses, which can serve as biomarkers of SLE progression and may contribute to disease propagation (3). The cGAS–STING pathway also plays a role in other autoimmune diseases, such as RA (4) and Sjögren syndrome (Ka et al.).

In response to chronic inflammation, the host attempts to limit tissue damage by producing anti-inflammatory cytokines, such as interleukin (IL)-37 and IL-38. These cytokines have been extensively studied in conditions such as osteoarthritis and RA (5), where they play a key role in mitigating the inflammatory environment (Zhang et al.). Specifically, IL-37 acts early by inhibiting pro-inflammatory pathways like STAT1, STAT3, p38 MAPK, ERK1/2, and NF-κB, and by influencing macrophage polarization, particularly the balance between pro-inflammatory (M1) and anti-inflammatory (M2) subsets. Conversely, IL-38 limits T helper 17 (Th17)-mediated response. Elevated levels of IL-37 and IL-38 have been found both systemically and locally in individuals with RA, and are linked to the Disease Activity Score (DAS) 28 and histological changes. As patients enter remission, the levels of IL-37 and IL-38 decline, suggesting their potential as biomarkers for disease progression and therapeutic response, as well as a guide for personalized therapy (6).

Liang et al. review how histone deacetylase 6 (HDAC6) modulates, at least in part, immune system functions and inflammatory responses in RA, SLE, and psoriasis.

### 2.1 The interaction between the immune system and microbial world

The bidirectional dialogue between the immune system and the microbial world continues to recalibrate our understanding of autoimmune diseases. Beyond its classical role in maintaining barrier integrity and hemostatic balance, the host–microbe interface has emerged as a potent modulator of systemic immunity. Microbial products can subvert tolerance through molecular mimicry, reshape lymphocyte repertoires, and activate pattern-recognition pathways that imprint long-lasting inflammatory tone. In this context, Wang et al. proposed gut-derived metabolites as discriminative biomarkers of immune status in RA. Through fecal metabolite profiling the authors distinguished between naïve and remission RA patients, implicating gut-derived bioactive compounds in systemic inflammatory modulation.

Converging evidence also underscores the importance of infectious triggers in autoinflammatory conditions such as synovitis, acne, pustulosis, hyperostosis and osteitis (SAPHO) syndrome (Wang et al.).

### 2.2 The relationship between the immune system and hemostasis

The intersection of immune activation, hemostatic regulation, and inflammatory signaling represents an increasingly recognized axis in the pathobiology of autoimmune and autoinflammatory disorders.

Sometimes immune and clotting responses can become simultaneously overly active, especially in diseases such as SLE, RA, and IBDs. Matsushita et al. reported a rare case of retroperitoneal fibrosis with IgG4-expressing plasma cell infiltration. The patient experienced recurrent life-threatening bleeding due to autoimmune coagulation factor XIII deficiency, which was successfully controlled through plasma exchange therapy. Mechanistic analysis revealed pathogenic autoantibodies targeting a structural component of the clotting factor, thereby impairing its function. This case underscores how immune dysregulation can directly undermine coagulation homeostasis, producing a dual burden of autoimmunity and hemorrhagic risk.

Despite being classically immune-mediated, a subset of patients remains unresponsive to immunosuppressive therapies. In this context, the report by Sau et al. is notable: the authors describe a case in which a thrombopoietin receptor agonist successfully restored platelet counts in a patient with immune thrombocytopenia resistant to multiple immunomodulatory regimens.

## 3 Biomarker-driven precision medicine in autoimmune and immune-mediated disorders

The accelerating shift toward precision medicine in immunology is grounded in the rapid expansion of molecular diagnostics capable of guiding individualized therapeutic decisions. By integrating genetic, serologic, and transcriptional data, clinicians are increasingly able not only to predict disease risk but also to tailor interventions that improve efficacy, minimize toxicity, and optimize healthcare resources. Autoantibodies remain among the most informative biomarkers in autoimmune and immune-mediated disorders. Liu et al. have illustrated this principle through an unusual anti-PL7–mediated syndrome in which a discrete serologic signature presaged unexpected pulmonary manifestations, with important therapeutic implications in the cure of early-stage disease. Similarly, the cases described by Qin and Yan have shown how convergent autoantibody profiles may unify multiple disparate neurological autoimmune conditions, underscoring the diagnostic and pathogenic value of humoral signatures.

Beyond serology, high-resolution transcriptional profiling is emerging as a powerful means of monitoring immune perturbations and therapeutic response. Royo et al. provide a compelling example in SLE, delineating the miRNA and mRNA alterations induced by belimumab treatment. Their findings support the potential of miRNAs, such as miR-146a, as novel biomarkers capable of capturing both disease activity and response to therapy.

Moreover, Xu et al. show that even therapeutics may be revitalized through biomarker-based stratification. They demonstrated that rituximab, despite its established role across multiple autoimmune diseases, can achieve improved cost-effectiveness and sustained clinical benefit when guided by individualized biomarker thresholds.

## 4 Advancing targeted therapies: from monoclonal antibodies to adoptive cell transfer

Over the past two decades, immunology has witnessed a decisive shift from broad immunosuppression toward precision manipulation of pathogenic pathways. Molecular-targeted agents, including JAK inhibitors and anticytokine monoclonal antibodies (mAbs), have redefined the standard of care in multiple autoimmune and inflammatory diseases by selectively extinguishing the signals that sustain chronic immune activation. The clinical success of TNF-α and IL-17 blockade in RA and psoriasis remains emblematic of this paradigm, demonstrating how interrupting a single cytokine axis can arrest the propagation phase of inflammation and induce durable remission. Indeed, the profound efficacy of TNF-α inhibition across RA and psoriatic arthritis underscores its role as a non-redundant driver of these disorders (7, 8).

Parallel advances in B-cell-targeted therapies have expanded the therapeutic armamentarium for antibody-mediated diseases. Deng et al. have highlighted the potential of ofatumumab, a novel fully human monoclonal antibody that selectively eliminates B lymphocytes, in anti-N-Methyl-D-aspartate receptors (NMDAR) encephalitis. They reported how next-generation mAbs may offer advantages in potency, tissue penetration, or safety relative to conventional B-cell–depleting agents.

Similarly, the landscape of Graves’ ophthalmopathy is rapidly evolving, with Wang and Chen providing a panoramic view of emerging targeted therapies (such as mAbs targeting CD20, IL-6 R, and insulin-like growth factor-1 receptor) aimed at interrupting fibroblast activation and orbital tissue remodeling.

Despite these advances, foundational challenges remain. Broad-spectrum immunosuppressants such as glucocorticoids and MTX remain first-line treatments reflecting both their therapeutic breadth and the persistent limitations of existing targeted options. Their systemic toxicities, however, underscore the urgent need for interventions that combine precision with durability. The advent of cell-directed immunotherapies, including CAR-T cells and Treg adoptive transfer, marks a new frontier in the treatment of refractory autoimmunity but requires further validation in clinical trials. For instance, CD19-targeted CAR-T therapy has achieved remission in SLE by eliminating autoreactive B cells (25). However, long-term safety and efficacy data are limited, and personalized approaches are essential to address disease heterogeneity. Future research should aim to identify biomarkers for early diagnosis and classify patients by molecular subtypes. Multiomics technologies and artificial intelligence may improve precision medicine by predicting treatment responses. Additionally, combination therapies, combining biologics with small-molecule inhibitors or microbiota modulation, may further enhance efficacy and reduce toxicity. Crucially, efforts to harmonize insights from preclinical models and clinical practice will be vital for developing curative strategies for autoimmune diseases.

## 5 Advancing autoimmunity research through integrative experimental and computational modeling

Pioneering studies on the autoimmune pathogenesis rely on the selection of well-characterized animal models able to capture the complex interplay of tolerance breakdown, effector responses, and tissue-specific inflammation. Liu et al. have described the reproducible generation of a murine model of autoimmune thyroiditis, providing the field with a valuable platform for dissecting the cellular and molecular mechanisms underlying thyroid-specific autoimmunity.

Zhang et al. have presented an elegant investigation into the therapeutic potential of DNase I in lupus nephritis using the MRL/lpr mouse model. They demonstrated that DNase I alleviates renal inflammatory injury by inhibiting the NETs/TLR4/MYD88 cell signaling axis, reducing the formation of NETs and the infiltration of immune inflammatory cells such as T cells and macrophages.

As computational power and multi-omic datasets continue to expand, in silico modeling is emerging as an indispensable counterpart to classical *in vivo* systems. Wei et al. have highlighted the utility of bioinformatic approaches in identifying shared pathogenic pathways across coexisting autoimmune diseases within the same individual. Importantly, their findings illustrated that integrative computational analyses can yield clinically actionable insights, enabling more precise and individualized therapeutic strategies.

## 6 The exploration of alternative therapies: natural compounds shaping B-cell and innate immune responses in autoimmune disease

Growing evidence suggests that nutraceuticals may confer therapeutic or preventive benefits across a variety of chronic conditions, including immune-mediated disorders. For instance, *Ganoderma lucidum*, a fungal product, component of traditional Chinese medicine, has garnered particular interest for its antioxidant and anti-inflammatory activities, and for the absence of reported toxicity (9). Huang et al. have revealed for the first time the critical role of neutrophils in the pathology of RA-related pain and demonstrated that *Ganoderma lucidum* effectively mitigated neutrophil-driven inflammation in a murine model of RA. Oral administration of *Ganoderma lucidum* spore powder promotes the transition of neutrophils in the paw tissues of collagen-induced arthritis mice from an N1 pro-inflammatory polarized state to an N2 anti-inflammatory polarized state, thereby exerting anti-inflammatory and analgesic effects.

Similarly, Rithvik et al. have shown that Habbe Gule Aakh (HGA), a traditional Unani polyherbal formulation composed of extracts from the flowers, has promising therapeutic potential in RA. HGA can rescue exacerbation of RA disease severity blocking TLR4 signaling axis and regulating the aberrant TLR4-mediated glycolytic flux in resident synoviocytes.

A study by Shimojima et al. has explored the effects of resveratrol on the expression of BAFF (B-cell activating factor) and APRIL (a proliferation-inducing ligand) in the context of ANCA-associated vasculitis (AAV). These molecules are well-known as survival and activating factors for B-cells, and their levels are increased in monocytes from patients with AAV. Therapies targeting BAFF, APRIL, and their receptors, such as belimumab and blisibimod (10, 11), represent potential treatment options. Belimumab shows therapeutic benefit when used alongside rituximab; however, when combined only with standard immunosuppressants, it does not prevent AAV relapse (12). These findings indicate that autoreactive B cells remain chronically stimulated in settings characterized by elevated BAFF and APRIL levels.

Shimojima et al. have revealed that resveratrol was able to modulate enhanced intracellular signaling of BAFF/APRIL production, potentially through redox reactions. Further investigations are necessary to clarify the precise intracellular signaling pathways that regulate BAFF/APRIL in all relevant immunocompetent cells that contribute to the development of AAV.

Furthermore, Heine et al. have demonstrated a potent synergy between omega-3 fatty acids and dexamethasone in the suppression of interferon-stimulated genes in macrophages of a mouse SLE model, supporting the therapeutic potential of dietary interventions in autoimmune conditions.

## 7 Conclusions

Collectively, the articles in this Research Topic deepen our understanding of autoimmune disease pathogenesis while illuminating novel and underexplored therapeutic avenues. They elucidate key mechanisms, define clinically meaningful biomarkers, and advance innovative strategies that hold promise for reshaping the diagnostic and therapeutic landscape.

The insights and emerging concepts presented here provide a strong foundation for future investigations aimed at targeting inflammation and immune dysregulation, and improving outcomes for patients affected by these complex and often refractory conditions.
